# Commensal Microbiota Enhance Both Osteoclast and Osteoblast Activities

**DOI:** 10.3390/molecules23071517

**Published:** 2018-06-23

**Authors:** Yoko Uchida, Koichiro Irie, Daiki Fukuhara, Kota Kataoka, Takako Hattori, Mitsuaki Ono, Daisuke Ekuni, Satoshi Kubota, Manabu Morita

**Affiliations:** 1 Department of Preventive Dentistry, Okayama University Graduate School of Medicine, Dentistry and Pharmaceutical Sciences, 2-5-1 Shikata-cho, Kita-ku, Okayama 700-8558, Japan; de20006@s.okayama-u.ac.jp (Y.U.); coichiro@md.okayama-u.ac.jp (K.I.); de20041@s.okayama-u.ac.jp (D.F.); de18017@s.okayama-u.ac.jp (K.K.); mmorita@md.okayama-u.ac.jp (M.M.); 2 Department of Microbiology and Immunology, Columbia University Medical Center, New York, NY 10032, USA; 3 Department of Biochemistry and Molecular Dentistry, Okayama University Graduate School of Medicine, Dentistry and Pharmaceutical Sciences, Okayama, Japan. 2-5-1 Shikata-cho, Kita-ku, Okayama 700-8525, Japan; hattorit@md.okayama-u.ac.jp (T.H.); kubota1@md.okayama-u.ac.jp (S.K.); 4 Department of Molecular Biology and Biochemistry, Okayama University Graduate School of Medicine, Dentistry and Pharmaceutical Sciences, 2-5-1 Shikata-cho, Kita-ku, Okayama 700-8558, Japan; mitsuaki@md.okayama-u.ac.jp; 5 Advanced Research Center for Oral and Craniofacial Sciences, Okayama University Dental School, Okayama, Japan 2-5-1 Shikata-cho, Kita-ku, Okayama 700-8558, Japan

**Keywords:** osteoblast, osteocalcin, Insulin-like growth factor-1, commensal microbiota, bone remodeling

## Abstract

Recent studies suggest that the commensal microbiota affects not only host energy metabolism and development of immunity but also bone remodeling by positive regulation of osteoclast activity. However, the mechanism of regulation of bone cells by the commensal microbiota has not been elucidated. In this study, 8-week-old specific pathogen-free (SPF) and germ-free (GF) mice were compared in terms of alveolar bones and primary osteoblasts isolated from calvarias. Micro-CT analysis showed that SPF mice had larger body size associated with lower bone mineral density and bone volume fraction in alveolar bones compared with GF mice. Greater numbers of osteoclasts in alveolar bone and higher serum levels of tartrate-resistant acid phosphatase 5b were observed in SPF mice. Tissue extracts from SPF alveolar bone showed higher levels of cathepsin K, indicating higher osteoclast activity. SPF alveolar extracts also showed elevated levels of γ-carboxylated glutamic acid–osteocalcin as a marker of mature osteoblasts compared with GF mice. Polymerase chain reaction (PCR) array analysis of RNA directly isolated from alveolar bone showed that in SPF mice, expression of mRNA of *osteocalcin*, which also acts as an inhibitor of bone mineralization, was strongly enhanced compared with GF mice. Cultured calvarial osteoblasts from SPF mice showed reduced mineralization but significantly enhanced expression of mRNAs of *osteocalcin, alkaline phosphatase, insulin-like growth factor-I/II*, and decreased ratio of *osteoprotegerin/receptor activator of nuclear factor-kappa B ligand* compared with GF mice. Furthermore, PCR array analyses of transcription factors in cultured calvarial osteoblasts showed strongly upregulated expression of *Forkhead box g1*. In contrast, *Gata-binding protein 3* was strongly downregulated in SPF osteoblasts. These results suggest that the commensal microbiota prevents excessive mineralization possibly by stimulating osteocalcin expression in osteoblasts, and enhances both osteoblast and osteoclast activity by regulating specific transcription factors.

## 1. Introduction

All mammals are exposed to environmental microorganisms, known as the commensal microbiota, after birth, and these organisms play important roles in maintaining homeostasis, such as in energy homeostasis [1] and innate immunity [2]. Recent studies suggest that the commensal microbiota can also influence bone remodeling [3,4,5,6].

Germ-free (GF) animals provide an invaluable experimental tool for examining interactions between a host and its microbiota. GF mice have hypoplastic Peyer’s patches, with reduced numbers of IgA-producing plasma cells and lamina propria CD4+ T cells [7]. Furthermore, GF mice have reduced adiposity and improved tolerance to glucose and insulin when compared to conventional specific pathogen-free (SPF) mice [8].

The term “osteomicrobiology” has recently been proposed by Ohlsson et al. [9] and Jones et al. [10] to refer to the role of the commensal microbiota in regulating postnatal skeletal development, bone development, bone aging, and pathologic bone loss. Bone metabolism, which is regulated by a bone turnover process that depends on the balance between osteoblasts and osteoclasts [11], is controlled by multiple systemic factors, including hormones, cytokines, and the immune system [12]. The effects of the immune system on bone remodeling have been investigated in recent studies generally by comparing SPF mice with GF mice [3,5,12]. GF mice established in the C57BL/6 strain have increased bone mass and density compared with SPF mice [3]. Furthermore, another report showed that the commensal microbiota exerts anti-anabolic effects (suppressing osteoblastogenesis) and pro-catabolic effects (enhancing osteoclastogenesis) in C57BL/6 mice [12]. Our group reported that SPF mice in the C3H/Orl strain exhibit increased numbers of osteoclasts per area of bone compared with GF mice [13]. Furthermore, histomorphometric analysis revealed increased alveolar bone loss in SPF mice compared with GF mice [13]. On the other hand, the SPF microbiota in the CB6F1 strain of mice increased bone formation rates and growth plate thickness [5]. The gut microbiota has pro-anabolic actions, enhancing liver insulin-like growth factor (IGF)-1–mediated skeletal growth in BALB/c and CB6F1 (mixed BALB/c and C57BL/6 background) mice [5,14]. The discrepancy in bone phenotype among previous studies is thought to be due to the potential influence of different mouse backgrounds on the osteoimmunoregulatory effects of the gut microbiota [3,5,12,14,15,16]. However, the details are not well understood.

Osteocalcin, a marker of osteoblasts, plays roles in bone formation and bone homeostasis. Osteocalcin can be carboxylated at glutamic acid residues (Glu) by vitamin K–dependent carboxylase and converted into γ-carboxylated osteocalcin (Gla-osteocalcin). Although Gla-osteocalcin is embedded in mineralized bone, osteocalcin knockout mice showed excessive mineralized bone formation. Therefore, osteocalcin was considered to inhibit bone mineralization [17]. Recent studies revealed that osteocalcin influences not only bones but also other organs [18,19]. Glu-osteocalcin regulates blood glucose levels by increasing insulin secretion and β-cell proliferation in the pancreas and by changing insulin sensitivity in adipose tissues, muscles, and liver [18]. Furthermore, osteocalcin induces testosterone production in Leydig cells of the testes [19]. Therefore, osteocalcin is now considered an important factor in the maintenance of whole-body homeostasis. Whereas Novince et al. reported higher expression of osteocalcin in osteoblasts from the femur in GF mice [12], Yan et al. reported higher expression of the *runx2* gene in epiphyseal bone in SPF mice [5]. Due to discrepancies in the literature, it is therefore unclear how the commensal microbiota affects osteoblast activity.

Based on the hypothesis that the commensal microbiota in SPF mice is involved in bone remodeling, the aim of this study was to investigate osteoclast and osteoblast activity in alveolar bones and cultured cells from 8-week-old GF and SPF mice (IQI/Jic). Here, we demonstrate that the presence or absence of the commensal microbiota affects expression of osteoblast-specific genes and transcription factors. These findings enhance our understanding of how the commensal microbiota functions in maintaining bone homeostasis.

## 2. Results

### 2.1. The Commensal Microbiota Increases Body Weight but Reduces Bone Mineral Density (BMD) in Alveolar Bone

**Figure 1:**
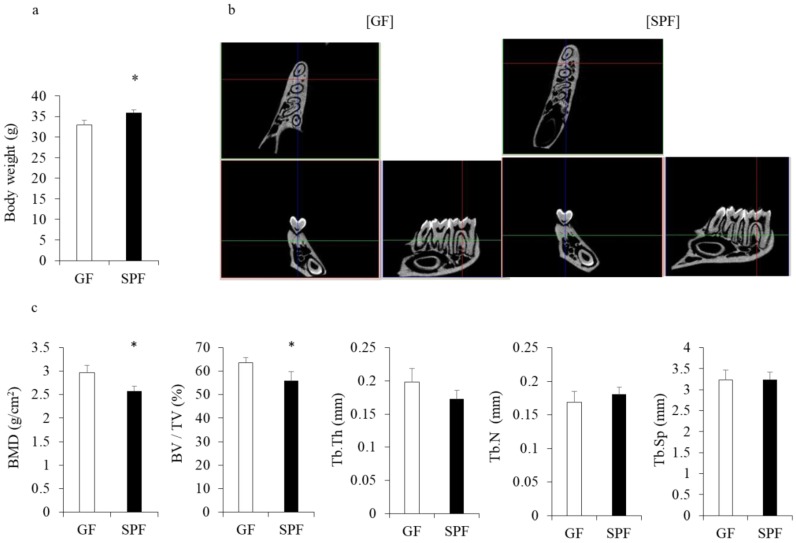
Animal weight and alveolar bone analysis. Eight-week-old male germ-free (GF) and specific pathogen-free (SPF) mice were weighed after isoflurane anesthesia, and then alveolar bones were harvested for micro- micro computed tomography (CT) analysis. (**a**) The weight of SPF mice was significantly greater than that of GF mice. * *p* < 0.05, *t*-test (*n* = 4/group). (**b**) 3D micro-CT image of root furcation area of the first molar. Left: GF; right: SPF. (**c**) Analysis of micro-CT volumetric parameters: bone mineral density (BMD), bone volume fraction (BV/TV), trabecular thickness (Tb.Th), trabecular number (Th.N), trabecular separation (Tb.Sp). * *p* < 0.05, *t*-test (*n* = 4/group).

Body weight (Figure 1a) and size (data not shown) were significantly greater in 8-week-old SPF mice than GF IQI/Jic mice (35.9 ± 0.655 g vs. 32.9 ± 1.097 g) (*p* < 0.05), in line with previous studies involving CB6F1 [6] and BALB/c mice [15]. To investigate whether the commensal microbiota affect microarchitectural changes in alveolar bone, we performed micro computed tomography (CT) analyses of alveolar bone and measured the root furcation area of the first molar (Figure 1b). Surprisingly, despite enhanced body weight, the BMD and bone volume fraction (BV/TV) of SPF mice were lower compared with GF mice (*p* < 0.05, Figure 1c), confirming results from Sjögren et al. [4] and our group [5] when analyzing long bones. No differences were observed in other microstructural parameters between GF and SPF mice (Figure 1c).

### 2.2. The Commensal Microbiota Enhances Osteoclast Differentiation

**Figure 2:**
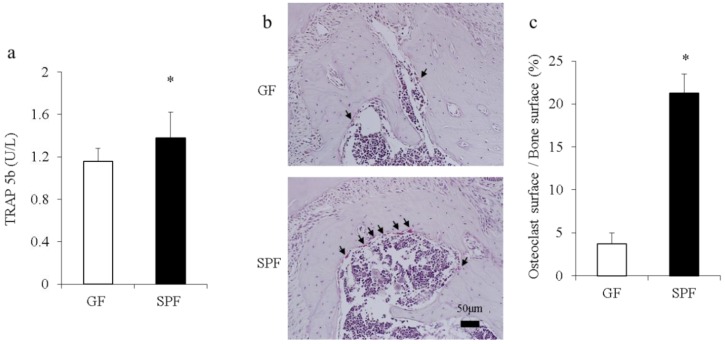
Serum levels of tartrate-resistant acid phosphatase (TRAP) and histomorphometric analysis of osteoclasts in GF and SPF mice. (**a**) Serum concentrations of bone markers from GF mice and SPF mice were determined by enzyme-linked immunosorbent assay (ELISA). The level of TRAP-5b was significantly higher in SPF mice than GF mice. * *p* < 0.05, *t*-test (*n* = 6/group). (**b**) Representative images of TRAP-stained spongiosa. Alveolar bones from 8-week-old GF (top) and SPF (bottom) male mice were harvested, and sagittal sections were analyzed by TRAP staining. TRAP-positive osteoclasts lining bone with ≥3 nuclei designating an osteoclast (arrows). Scale bar = 50 μm. (**c**) Osteoclasts and bone surface (μm) were measured in the range of 450 μm × 400 μm based on the alveolar crest using ImageJ computer software (version 1.45, NIH, Bethesda, USA). Comparable images and data are expressed as the ratio (%) of osteoclast surface/bone surface. Data are shown as mean ± SEM.

Considering changes in bone morphometric indices found in the remodeling alveolar bone of SPF mice (Figure 1b,c), serum and alveolar bone were collected from 8-week-old SPF and GF mice to examine how the commensal microbiota may affect osteoclastogenesis and activity. Serum levels of tartrate-resistant acid phosphatase (TRAP) 5b, a marker of osteoclast activity, and Gla-osteocalcin, a marker of osteoblasts, were analyzed by enzyme-linked immunosorbent assay (ELISA). Osteoclasts in alveolar bone mass were detected histomorphometrically by TRAP staining as an osteoclast cellular endpoint. The serum level of TRAP-5b was significantly higher in SPF mice than GF mice (*p* < 0.05) (Figure 2a). There was no significant change in the serum level of osteocalcin (data not shown). The osteoclast surface/bone surface ratio was significantly greater in SPF mice than GF mice (*p* < 0.05) (Figure 2b top: GF; bottom: SPF, and Figure 2c), suggesting greater osteoclastogenesis and osteoclast activity in SPF mice.

### 2.3. Levels of Ocn, Col1a 1, Ctsk, Mmp-2, and Mmp-9 mRNAs are Upregulated in SPF Alveolar Bones, and Serum Osteocalcin and Cathepsin K Levels are Increased

**Figure 3:**
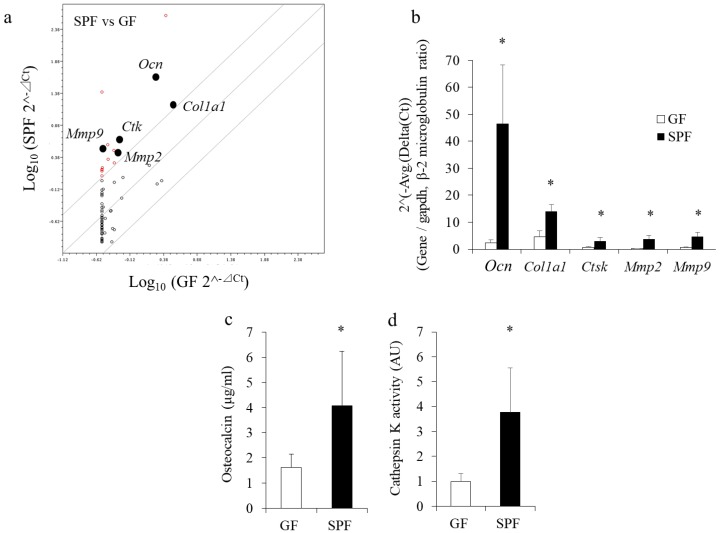
Enhanced expression of bone metabolism marker genes and proteins in SPF alveolar bone. Alveolar bone from 8-week-old GF and SPF mice was harvested for gene expression assays and ELISA. Total RNA isolated from alveolar bone was analyzed by polymerase chain reaction (PCR) array (**a**) and real-time PCR (**b**). (**a**) PCR array profiling of osteogenic gene expression in GF and SPF alveolar bone. Data from SPF (ordinate) and GF (abscissa) evaluations are plotted. Constantly elevated or varying levels of gene expression are indicated by red open circles. Circles on the diagonal line represent relative changes in metabolite levels that were stable between the two groups. (**b**) Closed circles in Figure 3a denote expression of genes that were significantly higher in SPF mice than GF mice (>4 times) examined in Figure 3b. (**c**,**d**) Alveolar bones from mandibles were manually homogenized, and proteins were extracted. Levels of γ-carboxylated glutamic acid–osteocalcin (**c**) and cathepsin K (**d**) were significantly higher in SPF mice than GF mice. * *p* < 0.05, *t*-test (*n* = 6/group).

To investigate changes in bone-specific gene expression profiles resulting from the presence of the commensal microbiota in SPF bone, total RNA was directly collected from SPF and GF alveolar bone and analyzed using polymerase chain reaction (PCR) microarrays. Among the genes examined, *osteocalcin* (*Ocn*)*, cathepsin K (Ctsk), matrix metalloproteinase (Mmp)-2, Mmp-9*, and *collagen type I alpha (Col1a) 1* mRNA levels were significantly higher in SPF mice than GF mice (*p* < 0.05) (Figure 3a). Changes in gene expression were confirmed by real-time PCR using total RNA from alveolar bones (Figure 3b). Protein extracts from alveolar bones of both SPF and GF mice were also collected and evaluated by ELISA. Levels of Gla- osteocalcin and cathepsin K were also significantly higher in SPF bones than GF bones (*p* < 0.05) (Figure 3c,d), suggesting that both osteoblastic and osteoclastic gene expression are higher in bones of SPF mice than GF mice.

### 2.4. SPF Osteoblasts Shows Less Calcification in Culture

**Figure 4:**
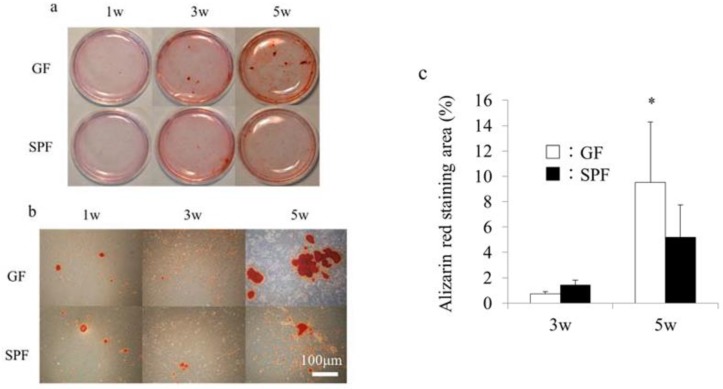
Calcification in cultured primary osteoblasts from germ-free (GF) and specific pathogen-free (SPF) mice. Calvariae from 8-week-old GF and SPF male mice were harvested, and primary osteoblasts were isolated and cultured in osteoblastic differentiation media. (**a**) Alizarin red staining of osteoblasts from GF and SPF calvariae cultured for 1, 3, and 5 weeks. Panel (**b**) shows high-magnification fields of panel (**a**). (**c**) The alizarin red–positive area was calculated using a computerized imaging system (ZEN, ZEISS, Oberkochen, Germany). The alizarin red–positive area at 5 weeks was significantly greater in SPF mice than GF mice. Mean values (±SD) are shown * *p* < 0.05, *t*-test (*n* = 4/group). Scale bar = 100 μm (**b**).

Our data indicated that the commensal microbiota enhanced both osteoclastic and osteoblastic gene expression in alveolar bone, in line with several reports showing that the commensal microbiota enhances osteoclast activity [3,5,11], although osteoblast activity was not examined in those studies. To investigate changes in osteoblast activity in vitro, primary osteoblasts were isolated from SPF and GF calvariae and cultured in osteoblastic differentiation medium. Calcification of extracellular matrix was examined by alizarin red staining at 1, 3, and 5 weeks of culture. At 1 and 3 weeks of culture, no significant differences in alizarin red staining were observed between GF and SPF mice (*p* > 0.05) (Figure 4a,b). However, at 5 weeks of culture, the alizarin red–positive calcified matrix area in osteoblast culture was significantly smaller in SPF mice than GF mice (*p* < 0.05) (Figure 4b,c). These results are in line with our micro-CT data (Figure 1), suggesting that the commensal microbiota prevents excessive mineralization possibly by stimulating osteocalcin expression in cultured calvarial osteoblasts.

### 2.5. Enhanced Expression of Opg, Rankl, Ocn, Alp, and Igf-I and II Genes in Long-Term Culture of SPF Osteoblasts

**Figure 5:**
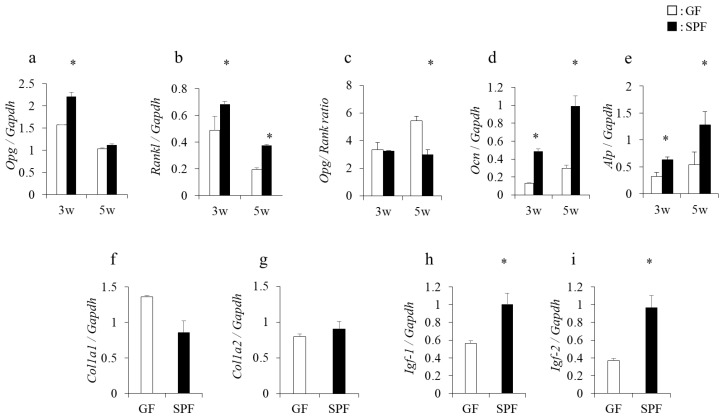
Osteogenic gene expression in calvarial primary osteoblasts. Calvariae from 8-week-old GF and SPF mice were harvested, and primary osteoblasts were isolated and cultured in osteoblast differentiation medium. After culture for the indicated times, total RNA was isolated, and real-time PCR analysis was performed to assess osteogenic gene expression. Significant differences in expression of the *osteoprotegerin* (*Opg*) gene (**a**), *receptor activator of nuclear factor-kappa B ligand* (*Rankl*) (**b**) gene, the *Opg/Rankl* ratio (**c**), *osteocalcin* (*Ocn*) (**d**) gene, and *alkaline phosphatase* (*Alp*) (**e**) gene were observed between GF and SPF osteoblasts. At 5 weeks of culture, no significant differences were observed in expression of the *collagen type I alpha* (*Col1a*) 1 (**f**) and *Col1a2* (**g**) genes between GF and SPF osteoblasts. Expression of the *insulin-like growth factor* (*Igf-1* (**h**) and *Igf-2* (**i**) genes was significantly higher in SPF osteoblasts than GF osteoblasts at 5 weeks. * *p* < 0.05, *t*-test (*n* = 4/group).

To investigate whether changes in gene expression levels seen in SPF alveolar bones also occur in cultured osteoblasts, total RNA was isolated from 3- and 5-week cultured osteoblasts, and gene expression was analyzed by real-time PCR. The expression of *osteoprotegerin* (*Opg*) mRNA at 3 weeks in SPF osteoblasts was significantly higher than in GF mice, but not at 5 weeks of culture (*p* < 0.05) (Figure 5a). In contrast, the expression of *receptor activator of nuclear factor-kappa B ligand* (*Rankl*) mRNA was significantly higher in SPF than GF osteoblasts at both 3 and 5 weeks of culture (*p* < 0.05) (Figure 5b), and the ratio of *Opg/Rankl* expression at 5 weeks was significantly lower in SPF than GF osteoblasts (*p* < 0.05) (Figure 5c). Furthermore, expression of *Ocn* and *alkaline phosphatase* (*Alp*) mRNA was significantly higher in SPF than GF osteoblasts (*p* < 0.05) (Figure 5d,e). However, no significant differences in the levels of *Col1a1* and *Col1a2* mRNAs between SPF mice and GF mice were observed (Figure 5f,g), but levels of *Igf-I* and *Igf-II* mRNAs were higher in SPF than GF osteoblasts cultured for 5 weeks (*p* < 0.05) (Figure 5h,i). These data indicate that osteoblast activity, including expression of growth-stimulating factors (*Igf-I* and *-II*) was enhanced in SPF-derived osteoblasts cultured for 3 and 5 weeks.

### 2.6. Gene Expression of Transcription Factors in Cultured Osteoblasts

**Figure 6:**
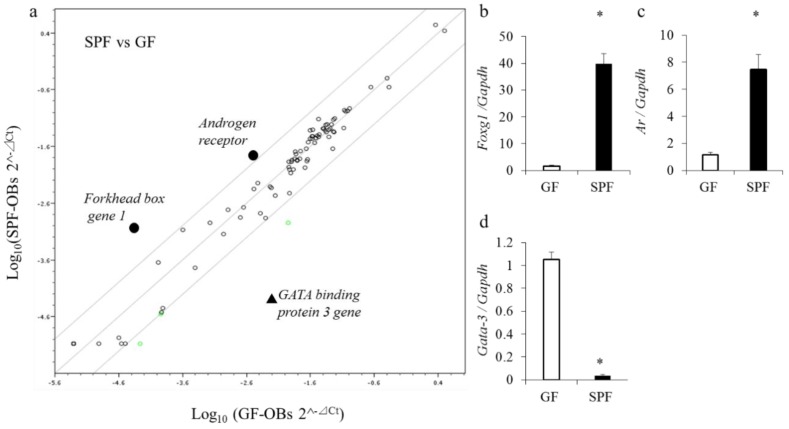
Gene expression profiling in cultured osteoblasts from GF and SPF mice. Calvariae from 8-week-old GF and SPF male mice were harvested, and osteoblasts were isolated and cultured. Total RNA was isolated for gene expression from osteoblasts cultured for 5 weeks. Microarray profiling of transcription gene expression in GF and SPF osteoblasts was performed (**a**). The data from the SPF (ordinate) and GF (abscissa) evaluations are plotted. Constant elevation or variation in levels of gene expression is indicated by black dots or the black triangle, respectively. Circles on the diagonal line represent relative changes in metabolite levels that were stable between the two groups. Black circles denote gene expression that was significantly higher in SPF mice than GF mice (>4 times). The black triangle denotes gene expression that was significantly lower in SPF mice than GF mice (>4 times). On real-time PCR, expression of the *forkhead box gene 1* (*Foxg1*) (**b**) and *androgen receptor* (*Ar*) (**c**) genes was significantly higher in SPF mice than GF mice. *Gata binding protein* (*Gata)-3* (**d**) gene expression was significantly lower in SPF mice than GF mice. * *p* < 0.05, *t*-test (*n* = 4/group).

To investigate the mechanisms that drive enhanced osteogenic gene expression in SPF osteoblasts, expression of transcription factors was analyzed by PCR array using osteoblast RNA. Three of the 84 genes on the array exhibited significant differences between GF mice and SPF mice (*p* < 0.05) (Figure 6a). To confirm the data from microarray analyses, real-time PCR analysis was performed and confirmed a significant induction of *forkhead box gene 1* (*Foxg1*) and *androgen receptor* (*Ar*) and a strong repression of *Gata binding protein* (*Gata*)*-3* in SPF osteoblasts as compared with GF osteoblasts (*p* < 0.05) (Figure 6b–d).

## 3. Discussion

In this study, we focused on bone morphometric and histomorphometric parameters and the expression of genes associated with bone metabolism in SPF mice compared with GF mice in vivo and in vitro. Whereas several studies have investigated effects on cultured osteoclasts [3,5], only one study has reported direct effects of the commensal microbiota on cultured osteoblasts [13] and showed anti-anabolic suppression of osteoblastogenesis in SPF mice. However, transcriptional regulation of bone metabolism by the commensal microbiota has not been reported.

First, we investigated the impact of the commensal microbiota on osteoclast activity. We detected significantly elevated serum levels of TRAP-5b and increased numbers of TRAP-positive osteoclasts in SPF mice. Alveolar bone from SPF mice showed enhanced expression of mRNAs for osteoclast markers such as *Ctsk*, *Mmp2*, and *Mmp9*. Furthermore, levels of cathepsin K protein, a marker of bone resorption that is highly expressed by osteoclasts [20,21], were significantly higher in SPF mice than GF mice. Our previous study also revealed that the number of TRAP-positive osteoclasts on the surface of alveolar bone was increased in SPF mice compared with GF mice [13].

Although we used a different mouse strain (IQI/Jic) here than in our previous study (C3H/Orl), the results of both were the same. Other studies using BALB/c, C57BL/6, and CB6F1 background mice described similar results regarding osteoclast activity in the presence or absence of the commensal microbiota [3,5,12]. Our results in all mouse strains examined strongly suggest that the presence of commensal microbiota is associated with accelerated activation of osteoclasts.

We also performed comprehensive gene expression analysis to determine the effect on osteoblast activity. Microarray profiling of osteogenic expression in alveolar bone revealed that the expression of osteoblast marker genes such as *Osteocalcin* and *Col1a1* was significantly higher in SPF alveolar bones compared with GF bones (*p* < 0.05, Figure 3). Osteocalcin is considered a specific marker of mature osteoblasts. The level of Gla-osteocalcin was also significantly higher in SPF than GF alveolar extracts. Osteoblasts isolated from SPF calvariae showed dramatically enhanced expression of osteocalcin in comparison with GF osteoblasts. This is in contrast to another recent study reporting that the commensal microbiota in C57BL/6 SPF mice suppressed osteocalcin mRNA expression in calvariae [12]. The reason for this discrepancy between the present and previous studies is unclear, but it may be related to differences in genetic background (IQI/Jic vs. C57BL/6), age (8 weeks vs. 11 weeks), experimental period (35 days vs. 21 days), and SPF condition (without fecal inoculum vs. with fecal inoculum).

Furthermore, we demonstrated that cultured SPF osteoblasts exhibit a lower *Opg/Rankl* ratio and enhanced expression of *Alp* related to differentiation of osteoblasts [22]. The present findings support those of previous studies indicating the normal gut microbiota has significant effects on both anabolic and catabolic activities in alveolar bone formation and physiologic skeletal growth [3,4].

Osteocalcin is an extracellular matrix protein in mature osteoblasts which is also important for the regulation of bone mineralization. For example, osteocalcin-knockout mice showed enhanced bone mineralization [17]. In this study, cultured osteoblasts showed significantly less mineralization in SPF mice than GF mice, in line with the higher expression of *Osteocalcin* mRNA in SPF osteoblasts. Accordingly, our Micro-CT analyses revealed that SPF alveolar bone had lower BMD and BV/TV compared with that of GF mice, probably due to a lower rate of mineralization. Thus, these data suggest that the commensal microbiota prevents excessive mineralization possibly by stimulating osteocalcin expression, which in turn controls bone remodeling.

IGF-1 produced by osteoclasts, osteoblasts, and osteocytes in an autocrine or paracrine manner plays an important role in bone remodeling [23,24,25,26,27]. IGF-1 can promote both bone formation and resorption through direct effects on osteoblasts [28,29,30,31]. In our cultured osteoblasts, the levels of *Igf-1* and *Igf-2* mRNAs were significantly higher in SPF mice than GF mice. The commensal microbiota reportedly increases insulin/IGF-like peptide activity in *Drosophila*, and IGF-1 was shown to mediate the microbiota’s effect on postnatal growth [32]. In BALB/c background mice, the gut microbiota was shown to enhance skeletal growth via upregulation of liver-derived IGF [14]. Our results suggest that the microbiota directly induces IGF-1 and -2 expression in osteoblasts, which supports postnatal growth. However, another previous study showed the opposite result using bone marrow and calvariae of C57BL/6 mice [12], indicating that expression of the *Igf-1* gene in bone marrow and calvariae in GF mice was higher than in SPF mice. Further studies are required to clarify this discrepancy.

The question arises as to how the presence of the commensal microbiota affects the expression of osteoblast-specific genes. The results of PCR array analyses of transcription factors in cultured osteoblasts of SPF mice showed substantial enhancement of the expression of *Foxg1* and *Ar* mRNA but strong repression of the expression of *Gata-3* mRNA in SPF osteoblasts. Foxg1, a member of the forkhead family of transcription factors, is essential for early brain development [33]. Foxg1 is also downstream of Igf-1 signaling [34]. A recent study revealed decreased expression of *osteocalcin* and *Alp* mRNAs in Foxg1-knockdown osteoblasts [35]. This is consistent with our finding that osteoblasts isolated from SPF calvariae showed enhanced expression of *osteocalcin* and *Alp* mRNAs in comparison to GF osteoblasts at 3 and 5 weeks, suggesting that the increase in *Foxg1* mRNA may be linked to upregulated expression of *osteocalcin* and *Alp*. Expression of FoxP3, a member of the Fox family and marker of regulatory T cells, is enhanced by the microbiota [36]. In osteoblasts, the commensal microbiota may effect expression of the *Foxg1* gene via a mechanism similar to that controlling the T-cell lineage.

The activation of Ar increases long bone growth and size [37]. In osteoblasts, it is critical for the suppression of androgen activity in osteoclastogenesis [38], but the Ar does not seem to suppress bone resorption through direct actions in osteoclasts [39]. Another study showed that the production of testosterone was reduced in GF mice [40]. In our study, the commensal microbiota may contribute to upregulation of androgen signaling by increasing Ar numbers in osteoblasts. Gata-3 is expressed mainly in hematopoietic cells and functions as a master regulator of T helper 2 (Th2) cell differentiation, regulating T-cell development, proliferation, and death [41]. Recent studies showed enhanced Th2-mediated immunity in GF mice, and Gata-3–positive cells were more numerous in GF mice than SPF mice [42]. Osteoblasts may be involved in directing T cell lineage toward Th1 and Th2 immune functions.

## 4. Materials and Methods

### 4.1. Mice

Male GF and SPF IQI/Jic mice were bred and maintained at the Central Institute of Experimental Animals (Kanagawa, Japan). GF mice (8 weeks old, *n* = 6) were housed in a Trexler-type flexible film isolator in a standard GF state and screened on a weekly basis for GF status by sterile feces sampling and culturing on MRS agar plates under aerobic and anaerobic conditions. SPF mice (8 weeks old, *n* = 6) were housed under SPF conditions. All mice were housed in an air-conditioned room (temperature 24 ± 1 °C) with a controlled light/dark cycle (lights on between 6:30 a.m. and 7:00 p.m.). Sterile food and water were available *ad libitum*. The rearing of the mice was conducted according to the institutional rules following approval from the Animal Experiment Committee of the Central Institute for Experimental Animals in Japan (16050A).

### 4.2. Micro-CT analyses

The mandibular bone was scanned using a desktop non-computed tomography system (Sky scan1174; Bruker Corporation, Billerica, MA, USA). The computed tomography settings were as follows: voltage, 50 mV; current, 800 kA; slice thickness, 7.4 μm. The images were reconstructed using Nreconc software (Bruker, Billerica, MA, USA). The area of septal bone of the first molar was measured using CTAn software (Bruker) according to the manufacturer’s instructions.

### 4.3. Serum ELISA

Whole blood was collected from the cardiac atrium under isoflurane anesthesia, and the serum was separated by centrifugation (4 °C, 10 min, 4000 g). The levels of TRAP-5b, a marker of bone resorption, were determined using a mouse TRAP Assay kit (IDS, Tyne and Wear, UK). Serum levels of osteocalcin, a marker of bone formation, were determined using a mouse Gla-osteocalcin high-sensitivity EIA kit (TAKARA BIO Inc., Shiga, Japan). Absorbance was measured by microplate reader at 405 nm for the TRAP Assay and at 450 nm for Gla-osteocalcin (SH-1000, Corona, Ibaragi, Japan). Both assays were performed according to the manufacturers’ instructions. All serum samples were assayed in duplicate.

### 4.4. TRAP Staining

The left alveolar arches of the maxillae were resected en bloc and fixed in 4% paraformaldehyde, 0.1 M phosphate buffer (pH 7.4) for 24 h, followed by decalcification in 10% tetrasodium ethylenediaminetetraacetic acid aqueous solution (pH 7.4) for 2 weeks at 4 °C. The samples were dehydrated and embedded in paraffin, then 4-μm-thick serial sections were obtained through the mesial-distal width of the tooth. To detect TRAP-positive osteoclasts using azo dye methods, sections were counterstained with Mayer’s hematoxylin. Images were acquired using an IX70 microscope (Olympus, Tokyo, Japan) [43,44]. The osteoclast surface/bone surface was measured in the range of 450 μm × 400 μm based on the alveolar crest using ImageJ computer software (NIH).

### 4.5. Alveolar Bone Homogenates

Alveolar bones from mandibles were manually homogenized using a frozen cell crusher (Microtec Co., Chiba, Japan) in T-PER reagent (Pierce, Rockford, IL, USA) containing a protease inhibitor mixture (Roche Applied Science, Penzberg, Germany). Total protein concentration was determined by Bradford assay (Bio-Rad, Hercules, CA, USA). Osteocalcin (TAKARA BIO Inc., Shiga, Japan) and cathepsin K (Cathepsin K Activity Assay kit, Abcam, Cambridge, UK) levels were measured using commercial kits. Concentrations were calculated using standard curves following the manufacturers’ protocols [45,46]. All bone samples were assayed in duplicate.

### 4.6. Calvarial Osteoblast Culture

After removal of sutures, calvariae were subjected to a series of collagenase digestions for 30 min at 37 °C on a rocking platform. The first two digests were discarded. The third digest was collected, and enzyme activity was stopped by addition of an equal volume of αMEM (Invitrogen, Carlsbad, CA, USA) containing 10% fetal bovine serum (FBS) (Hyclone, SH30070.03), 100 U/mL of penicillin, and 100 mg/mL of streptomycin (Gibco BRL, Grand Island, NY, USA). Cells were harvested at a density of 3.5 × 10^4^ cells/cm^2^ in a 3.5-cm dish in αMEM containing 10% heat-inactivated FBS. At 3 days, the medium was changed. After 1 week, the medium was changed to differentiation medium (αMEM containing 10% FBS, 50 mg/mL of ascorbic acid, and 4 mol/mL of beta-glycerophosphate) [47,48,49]. The medium changes were performed every 3 days. The cells were collected and used for experiments with 1-, 3- and 5-week cultures.

### 4.7. Alizarin Red Staining

Cells were washed with PBS and fixed with 100% ethanol for 5 min at room temperature. After washing cells with PBS, alizarin red staining solution (pH 6.4 with 28% ammonium hydroxide) was added to the wells and incubated at room temperature for 15 min. Cells were washed with PBS. Images were acquired using an IX70 microscope (Olympus, Tokyo, Japan) [44]. Mineralized nodules were visualized, and the staining area was measured using a computerized imaging system (ZEN, ZEISS) at 1, 3, and 5 weeks of culture. Each measurement was repeated three times.

### 4.8. RNA Preparation and PCR Array

Total RNA was isolated from the alveolar bone and osteoblast cell samples using Trizol reagent (Invitrogen), in accordance with the manufacturers’ instructions. Isolated RNA was quantified by measuring the absorbance at 260 nm, and purity was determined by the 260/280 nm absorbance ratio. Comprehensive gene expression assays were performed using the 96-well RT^2^ Profiler^TM^ PCR Array system (SA Biosciences, Frederick, MD, USA) for the mouse osteogenesis (PAMM-026Z) and mouse transcription factor (PAMM-072Z) arrays in accordance with the manufacturer’s instructions. Thermal cycling was performed using a StepOnePlus Real-Time PCR System (Applied Biosystems, Foster City, CA, USA) with an initial denaturation at 95 °C for 10 min, 40 cycles at 95 °C for 15 s, and 60 °C for 1 min. A signal was acquired at 60 °C during each cycle. Values of the cycle threshold (Ct) obtained in quantification were used for calculations of fold changes in mRNA abundance using the 2^−ΔΔCt^ method [50].

### 4.9. Real-Time Quantitative PCR

Total RNA was reverse-transcribed using a PrimeScript™ RT reagent kit (Takara Bio Inc., Shiga, Japan) at 35 °C for 15 min and 87 °C for 1 min. Real-time PCR was performed using SYBR Green Real-time PCR Master Mix (Toyobo, Osaka, Japan) in a StepOnePlus Real-Time PCR System (Applied Biosystems, Foster City, CA, USA). Primer sequences are shown in Table 1. The amplification conditions were as follows: 40 cycles at 95 °C (10 s), 64 °C (10 s), and 72 °C (15 s). The mRNA levels were calculated in terms of the relative copy number ratio of each mRNA to *Gapdh* for each sample [43,51].

### 4.10. Statistical Analysis

Data are presented as means ± standard deviation. Student’s two-tailed unpaired *t*-tests were used for comparisons between GF and SPF mice to determine whether there were any significant differences. All calculations were performed using the statistical software package SPSS 22.0 for Windows (SPSS Japan, Tokyo, Japan); *p* < 0.05 was considered significant [51].

## 5. Conclusions

We demonstrated that the presence of the commensal microbiota prevents excessive mineralization possibly by stimulating osteocalcin expression in osteoblasts, and enhances the activity of both osteoblasts and osteoclasts. The balance between osteoblasts and osteoclasts may explain discrepancies in bony phenotypes in different strains of SPF and GF mice. The commensal microbiota plays an important role in directing the activity of osteoblasts and osteoclasts by regulating upstream transcription factors.

## Figures and Tables

**Table 1:** Primers used for real-time PCR analysis.

Gene	F/R	Primer Sequences (5′–3′)
*Opg*	F	Gttcctgcacagcttcacaa
	R	Aaacagcccagtgaccattc
*Rankl*	F	Gaactgcaacacattgtggg
	R	Attgatggtgaggtgtgcaa
*Ocn*	F	Gagtctgacaaagccttca
	R	Agccatactggtctgatag
*Alp*	F	Gtgccagagaaagagagagac
	R	Gacgcccataccatctcc
*Col1a1*	F	Agttggtgctaagggtgaag
	R	Gcaataccaggagcaccatt
*Col1a2*	F	Ctgatggcagagctggtgta
	R	Atgttgccagcttcacctct
*Igf-1*	F	Gtgtggaccgaggggcttttacttc
	R	Gcttcagtggggcacagtacatctc
*Igf-2*	F	Gtggcatcgtggaagagtgc
	R	Ggggtgggtaaggagaaacc
*Foxg1*	F	Gtgatgctggacatgggaga
	R	Gtggtggttgtcgttctgga
*Ar*	F	Cagcagcataccagaatcgc
	R	Tccaatgggttctccagctt
*Gata-3*	F	Cctaccgggttcggatgtaa
	R	Atggtagagtccgcaggcat
*Gapdh*	F	Tgtgatgggtgtgaaccacgagaa
	R	Gagcccttccacaatgccaaagtt

## Abbreviations

GF	Germ-free
SPF	Conventional specific pathogen-free
TRAP-5b	Tartrate-resistant acid phosphatase-5b
IGF-1	Insulin-like growth factor-1
Mmp-2	Matrix metalloproteinase-2
Ocn	Osteocalcin
Alp	Alkaline phosphatase
Mmp-9	Matrix metalloproteinase-9
Foxg1	Forkhead box g1
Ar	Androgen receptor
Gata3	Gata-binding protein 3
PCR	Polymerase chain reaction
Micro-CT	micro-computed tomography
ELISA	enzyme-linked immunosorbent assay
